# Functional Outcomes After the Sacrifice of Mandibular Condyle Using Fibula Free Flap for Immediate Surgical Reconstruction

**DOI:** 10.7759/cureus.60103

**Published:** 2024-05-11

**Authors:** Ashton L Rogers, Soroush Farsi, Noah Slater, James R Gardner, Deanne King, Jumin Sunde, Emre Vural, Mauricio Moreno

**Affiliations:** 1 Department of Otolaryngology - Head and Neck Surgery, University of Arkansas for Medical Sciences, Little Rock, USA

**Keywords:** head and neck cancer, condyle reconstruction, neo-condyle, mandible condyle, fibula free flap

## Abstract

Introduction

Head and neck cancer with mandibular invasion often necessitates composite resection, leading to defects requiring reconstruction. Microvascular fibula free flap (FFF) surgery is a common approach for this purpose. In this study, we focus on our experience with condyle sacrifice, emphasizing treatment outcomes and functional results. Additionally, we highlight a contemporary perspective by discussing surgical techniques and radiographic outcomes based on a 3D analysis of neo-condyle placement on CT imaging.

Methods

We studied 23 patients who had undergone segmental mandibulectomy requiring FFF reconstruction between 2009 and 2020. These were all performed by the same surgeon (M.M.) at an academic tertiary care center. Twenty-three reconstructions included condyle sacrifice. Retrospective chart review was performed with a focus on treatment, functional outcomes, and surgical technique.

Results

A total of 23 patients were included in the study group (13 females and 10 males) with a mean age of 58.1 years. The most common surgical indication was for oncologic purposes (n=9; 39.1%). Twenty (87%) patients required tracheostomy, and all were decannulated. In terms of surgical complications, two (8.7%) patients had a degree of arterial insufficiency and two (8.7%) developed delayed infections. The average inpatient stay was 5.61 days, with a subsequent average clinic follow-up after 16.9 days. CT or MRI imaging was available for 21 (91.3%) patients, showing 14 (66.7%) neo-condyles within the glenoid fossa. Fifteen (71.4%) patients had some element of anterior displacement (average=6.27 mm), and seven (33.3%) patients had a component of lateral displacement (average=2.23 mm). Three (13%) patients died during the follow-up period. Eighteen (90%) of the surviving patients returned to an oral diet within an average of 24.9 days. All patients returned to normal interincisal distance by 12 months. All FFFs, with and without complications, remained viable.

Conclusion

We achieved favorable oral function outcomes in the majority of our patients. Intriguingly, although radiographic evidence revealed anterior and/or lateral displacement of the neo-condyle, there was no observed correlation with the resumption of oral diet, trismus, or crossbite among these patients.

## Introduction

Head and neck cancer (HNC) ranks as the seventh most prevalent type of cancer worldwide and encompasses a heterogeneous spectrum of malignancies originating from the anatomic regions including the upper aerodigestive tract [1,2]. HNC accounts for around 4% of all cancers in the United States. In 2023, an estimated 66,920 people (49,190 men and 17,730 women) were diagnosed with HNC [3]. Head and neck squamous cell carcinomas (HNSCCs) constitute the majority, around 90%, of all HNC cases and can manifest in various anatomical locations [4]. While approximately 75% of HNSCC cases are linked to tobacco and alcohol use, a minority (30%) are attributed to human papillomavirus (HPV) infection [5-8]. In the United States, the decrease in cigarette consumption has been associated with a reduction in the overall incidence of HNSCC, except for HPV-related (HPV+) oropharyngeal squamous cell carcinoma (OPSCC), which is notably increasing in frequency [9].

Patients with head and neck lesions involving the mandibular condyle, ramus, body, and/or symphysis requiring mandibular resection can be a clinical challenge for head and neck surgeons. Often, the disease process is advanced, encompassing invasion or destruction of the mandibular cortex as well as surrounding cutaneous and/or mucosal soft tissue. With advanced disease, a more conservative resection, in the form of marginal mandibulectomy, is no longer considered secondary to poor local control and thus will typically require a segmental mandibulectomy. When the disease process involves or approaches the condylar head, a dilemma arises between preserving the native condyle for functional purposes or performing condyle resection for oncologic margin purposes [1,2]. Some subscribe to keeping a condyle remnant, if possible, but this leaves a questionable blood supply to the fragment, and the remnant may not be suitable for plating during the reconstructive portion [3,4]. A complete condyle defect with adjacent mandible resection thus presents a reconstructive challenge to restore temporomandibular joint function.

The literature presents many options for the reconstruction of the temporomandibular joint when condyle sacrifice is necessary [10-12]. From a historical perspective, many of these patients were previously left without reconstruction in a “mandibular swing.” Reconstruction of smaller defects can be considered with grafting options of autologous costochondral or calvarial bone, but they have the disadvantage of poor blood supply in a patient likely requiring adjunctive radiation therapy. Prosthetic materials have been used for condyle replacement, but the increased risk of glenoid fossa erosion into the middle cranial fossa and foreign body reactions have tapered expectations. Microvascular fibula free flap (FFF) techniques have established themselves as an increasingly viable reconstructive option for mandibular defects. They meet the criteria of having excellent blood supply to withstand adjunctive therapies, ample material for various defect sizes, the ability to tailor to specific defect sites, and are autologous. The support of condyle reconstruction with FFF transfer has been explored within the surgical community; early apprehensions included ankylosis and trismus with poor functional outcomes with variable cosmetic results [13,14]. More recent work suggests the contrary, that is, patients can expect restoration of function and good cosmetic results [15,16]. We present our condyle sacrifice experience with treatment and function but with a more contemporary focus on surgical technique and radiographic outcomes, specifically using CT imaging for 3D analysis of neo-condyle placement.

## Materials and methods

The study was approved by the Institutional Review Board of the University of Arkansas for Medical Sciences, Little Rock, AR, USA (IRB# 228942). The data were collected retrospectively from the departmental database, which was queried to identify 206 patients who underwent segmental with subsequent FFF reconstruction between September 2009 and June 2020. Within this cohort, 23 were identified to have had condylar head sacrifice. All of the cases were performed by the senior author (M.M.), and no cases were excluded from the series. The information retrieved from the medical records included demographics, surgical indications, radiation exposure, flap outcomes, length of hospital stay, postoperative complications, and average clinic follow-up. Functional outcomes gleaned from medical records included degree of trismus, presence of open bite deformity, time to resumption of oral diet, and tracheostomy decannulation rates. Operative report descriptions and intraoperative photography were reviewed to document the location and extent of the defect along with flap composition and size. The surgical technique review was focused on defect size, post-operative imaging, and condyle remodeling. Post-operative imaging was obtained using computed tomography (CT) or magnetic resonance imaging (MRI) typically within 6-12 months of initial surgery. These were reviewed to identify neo-condyle head placement within the glenoid fossa, lateral and anterior displacement, and evidence of neo-condyle remodeling. Distance from the glenoid fossa was measured to establish a quantitative measure in lateral and anterior dimensions. The anterior cut-off criterion was 7.5 mm, and the lateral cut-off criterion was 5 mm.

All patients underwent a preoperative videofluoroscopic swallowing study (VFSS) to obtain baseline swallowing function and repeat VFSS on postoperative clinic follow-up within three weeks. At our institution, patients undergoing free flap reconstruction of the oral cavity or mandible typically maintain strict NPO (nil per os) for two to three weeks, followed by a formal VFSS to determine if they can resume oral diet. Aggressive swallowing therapy encouraging oral diet is initiated at this stage, regardless of the use of adjuvant therapy. Patients were instructed to begin passive mobilization exercises upon inpatient discharge. If within the initial postoperative clinic visits, severe or moderate trismus was identified, they were instructed to begin active jaw mobilization with a rehabilitation system, TheraBite system.

Surgical technique

A tracheostomy was performed at the initiation of the procedure. Exposure was provided via lip-splitting incision when increased access was required for oncologic resection. Patients with benign pathology and favorable access required only a cervical incision with soft tissue flap elevation to free the pathologic segment of the mandible. The neck was explored for candidate vessels in anticipation of future reconstructive anastomosis. Hemimandibulectomy was performed with wide margins for complete tumor removal, including the condylar head. The contour of the mandible was replicated with a prefabricated plate template prior to resection, when possible, and if this was not possible secondary to tumor burden, the patient was placed into mandibular maxillary fixation after resection to help approximate mandibular contour. Donor fibula harvest was undertaken based on magnetic resonance angiography (MRA) of the lower extremity vessel runoff, and the contralateral fibula was favored for its anatomic relationship in the vascular pedicle and skin paddle inset. Osteotomies were performed on the resected fibula and plated to help replicate the mandibular angle contour and creation of neo-condylar head; several centimeters of fibula were left superior to the plate to allow for fitting of neo-condyle into the glenoid fossa. No reconstruction of the glenoid fossa was performed, and resection of the articular disk was not performed in this series. A drill was used to create a pilot hole through the neo-condyle head, and a securing 2.0 Prolene suture was threaded through the pilot hole and subsequently anchored to the anterior lip of the glenoid fossa. Microvascular anastomosis of peroneal vessels was then performed on recipient cervical neck vessels.

## Results

A total of 23 patients were included in the study group (13 female and 10 male patients) with a mean age of 58.1 years (range: 31-78 years). The most common surgical indication was for oncologic purposes (n=9; 39.1%), including resection of squamous cell carcinoma (n=7; 30.4%), sarcoma (n=1; 4.3%), and mucoepidermoid carcinoma (n=1; 4.3%). There were five (21.7%) patients with osteoradionecrosis. The remaining patients had ameloblastoma (n=4; 17.4%), osteochondroma (n=1; 4.3%), odontogenic cyst (n=1; 4.3%), and vascular malformation (n=1; 4.3%). Nine (39.1%) patients had a prior history of radiation therapy to the head and neck region.

The mean mandible defect size was 10.75 cm (range: 2.5-14 cm). Twelve (52.1%) patients had an oral cavity defect requiring an additional soft tissue flap with an average size of 75.58 cm2 (range: 10-180 cm2). Four (17.4%) patients required partial glossectomy resection. Twenty (87%) patients required tracheostomy; all were decannulated. The average inpatient stay was 5.61 days. Factors contributing to prolonged inpatient stay were ileus, delirium, and infection. In terms of surgical complications, two (8.7%) patients had arterial insufficiency: arterial thrombosis requiring revision anastomosis on postoperative day (POD) 0 and hematoma on POD 12. In addition, two (8.7%) patients developed delayed infections requiring hardware removal. All FFFs, with and without complications, remained viable.

**Figure 1:**
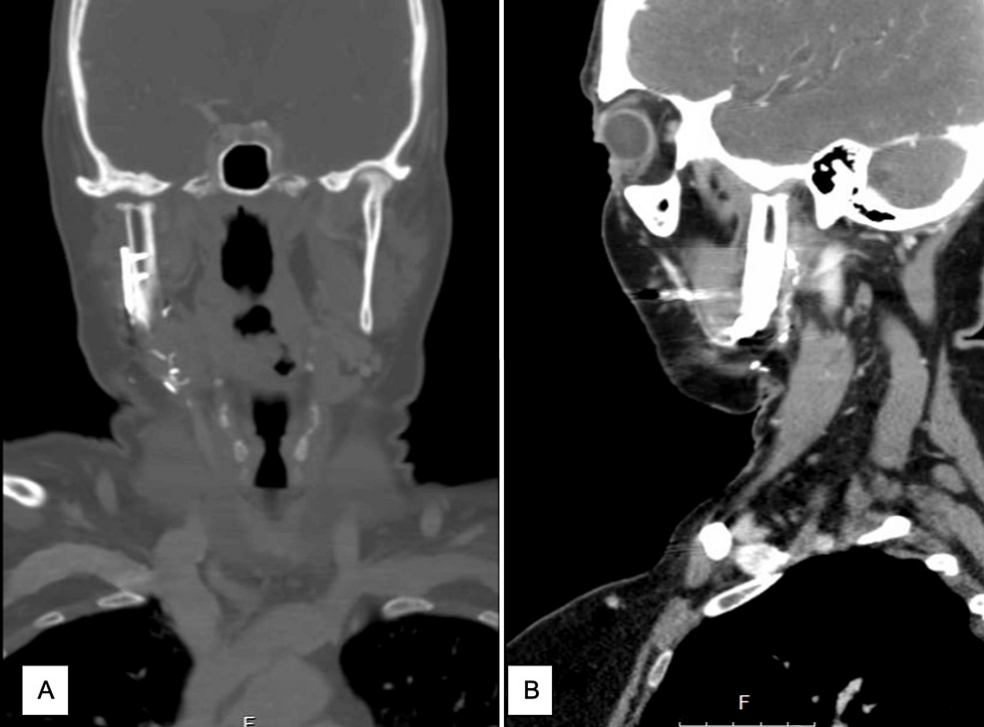
CT imaging of neo-condyle within the glenoid fossa in coronal view (A) and sagittal view (B).

**Figure 2:**
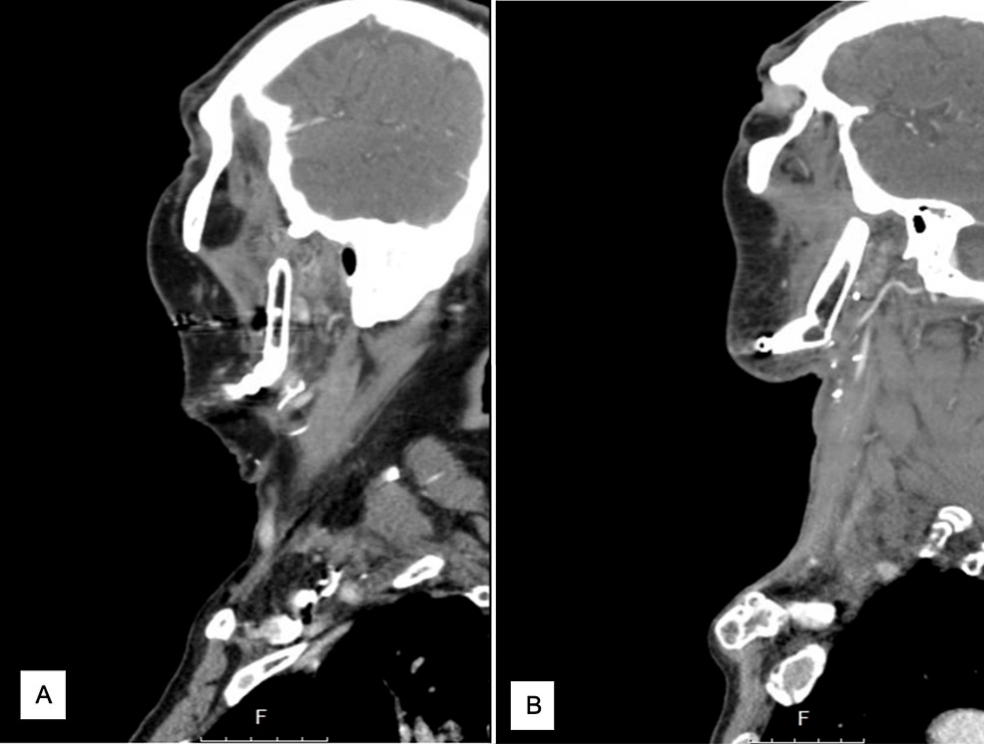
CT imaging of anterior displacement greater than 7.5 mm in two sagittal views (A and B).

**Figure 3:**
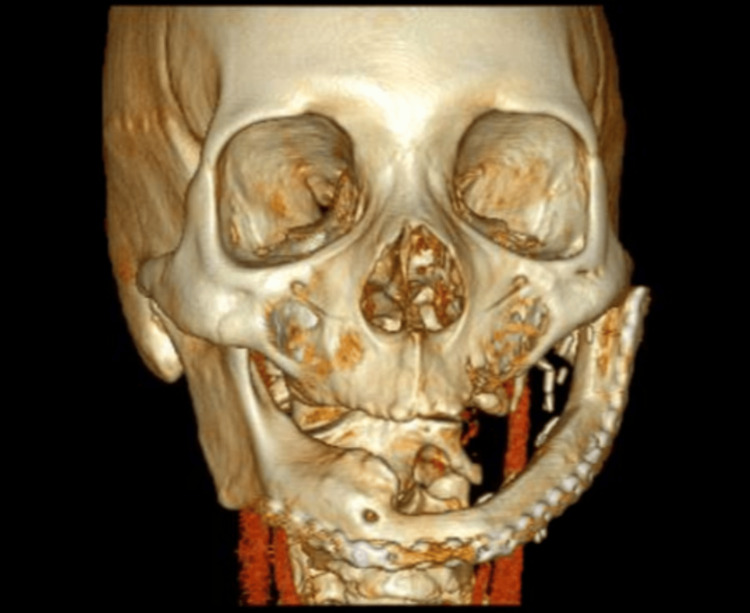
A 3D CT of lateral displacement greater than 5 mm.

Twenty-one (91.3%) patients had CT or MRI imaging available in the 12-month postoperative period (Figures 1-3). This revealed that 14 (66.7%) neo-condyles were within the glenoid fossa. Fifteen (71.4%) patients had some element of anterior displacement (average=6.27 mm), and seven (33.3%) patients had a component of lateral displacement (average=2.23 mm). The average postoperative visit was 16.9 days after surgery. Most patients denied trismus (n=12; 57.1%) at that time. However, two (9.5%) patients reported having severe trismus, one (4.8%) had moderate trismus, and five (23.8%) had minimal trismus (Table 1).

**Table 1:** Patients' radiographic glenoid fossa alignment, anterior and/or lateral displacement measurements, degree of trismus at POV, and resumption of oral diet. POV, postoperative visit

Patient	Within the glenoid fossa	Anterior displacement (mm)	Lateral displacement (mm)	Trismus	Resumption of oral diet
1	Yes	5	4	Minimal	No
2	No	8.5	0	None	No
3	No	14.6	0	None	Yes
4	No	10	5	None	Yes
5	No	8.3	10	None	Yes
6	No	9.1	0	Severe	Yes
7	Yes	6.3	0	Severe	Yes
8	Yes	7.5	0	Minimal	Yes
9	Yes	0	0	Moderate	Yes
10	Yes	7.5	0	None	Yes
11	Yes	13.5	4.2	Minimal	Yes
12	Yes	8.7	0	None	Yes
13	No	3.1	2.6	None	Yes
14	Yes	2.8	1.1	Minimal	Yes
15	Yes	0	1	Moderate	Yes
16	Yes	0	0	None	Yes
17	No	5.5	0	Minimal	Yes
18	Yes	0	0	None	Yes
19	Yes	0	0	None	No
20	Yes	4	0	None	Yes
21	Yes	0	0	None	Yes

Of the 15 patients with some degree of neo-condyle displacement, three (20%) returned to an oral diet despite developing a crossbite. The patients who developed crossbite also lacked neo-condyle alignment with the glenoid fossa. Three (20%) patients with anterior, lateral, and glenoid fossa displacement did not develop crossbite and successfully returned to an oral diet. The majority were cleared for an oral diet and resumed an oral diet postoperatively (n=13; 86.7%) (Table 2). The average return to oral diet was 24.9 days (range: 9-74 days). All patients returned to a normal interincisal distance (>40 mm) by 12 months.

**Table 2:** Relationship of neo-condyle displacement with trismus at POV, crossbite, and resumption of oral diet. POV, postoperative visit; (+) Anterior, presence of anterior displacement; (+) Ant/Lateral, presence of anterior and lateral displacement; (+) Glenoid, neo-condyle meets criteria for glenoid fossa alignment; (-) Glenoid, neo-condyle does not meet criteria for glenoid fossa alignment

	Trismus POV	Crossbite	Oral Diet Resumption
(+) Anterior (+) Glenoid (n=5)	None	Yes	Yes
	None	No	Yes
	None	No	Yes
	Minimal	No	Yes
	Severe	Yes	Yes
(+) Anterior (-) Glenoid (n=4)	None	No	No
	None	No	Yes
	Minimal	No	Yes
	Severe	No	Yes
(+) Ant/Lateral (+) Glenoid (n=3)	Minimal	No	No
	Minimal	Yes	Yes
	Minimal	No	Yes
(+) Ant/Lateral (-) Glenoid (n=3)	None	No	Yes
	None	No	Yes
	None	No	Yes

Three (13%) patients died during the follow-up period: one passed in the immediate postoperative period (POD 10) at the acute rehab facility, one patient succumbed during chemotherapy/radiation therapy, and one patient succumbed secondary to disease recurrence at postoperative month 18. Removing the three patients who died within the postoperative period, 18 (90%) of the 20 surviving patients resumed an oral diet. The two (10%) patients who did not return to an oral diet were gastrostomy tube (G-tube) dependent prior to surgery without beneficial change postoperatively. Six (30%) of the surviving patients, not already G-tube dependent, required G-tube feeds postoperatively for supplemental nutrition.

## Discussion

Use of microvascular FFF for reconstruction of advanced-stage disease with subsequent complex mandibular defects is an increasingly viable option for the head and neck surgeon [17]. In cases of disease involvement including condyle and subsequent condyle removal, the FFF presents a reliable tool for temporomandibular joint reconstruction. Maurer et al. presented their experience with 15 patients and reported that all patients had favorable functional status of mouth opening, reduction of pain, mouth excursions, and oral intake; any radiologic deviation of the neo-condyle was not believed to diminish clinical function [15]. They agreed that preservation of the native condyle was not necessary for effective temporomandibular joint function if reconstructed with FFF secured in place to the anterior lip of the glenoid fossa.

Other options for securing the neo-condyle within the fossa include suturing the remaining masseter muscle to the pterygoid muscles to form a sling [10,18]. Swendseid et al. in a series of 21 patients with condyle reconstructions found that 83.3% of these patients returned to a normal diet and denied any dysphagia, and while slight radiographic displacement may be noted, gross condyle migration is unlikely [19]. Eleven of the patients had postoperative CT scans assessing the reconstruction site; the condyle location was stable in all patients. Yu et al. appreciated a positive relationship between neo-condyle regeneration postoperatively and shorter stable mean time as well as stable position of the neo-condyle in relation to the glenoid fossa during this phase. They also observed long-term success of the mandibular condyle using a fibular flap correlated to variables including age, gender, and number of fibular segments [20]. Another study by Gilliot et al. found disc preservation as an important factor but other contributing factors toward the quality of result, as age, previous surgery performed, and previous radiotherapy [21].

Seth et al. from the Cleveland Clinic introduced a novel method of neo-condyle placement within the glenoid fossa. Using a miniplate secured to the root of the zygomatic arch, a Prolene suture was passed through the miniplate to the neo-condyle head. All three patients had confirmed placement within the glenoid fossa on radiographic review using CT imaging; additionally, patients reported improved facial symmetry, jaw opening, and acceptable dental occlusion without joint ankylosis [22].

Our study is the first to combine a cohort of 23 patients requiring condyle reconstruction with microvascular FFF focusing on functional outcomes but also using a contemporary radiographic analysis of neo-condyle placement using 3D CT or MRI versus 2D Panorex films. Fifteen (71.4%) patients had some element of anterior displacement (average=6.27 mm), and seven (33.3%) patients had a component of lateral displacement (average=2.23 mm). We expected a certain degree of anterior displacement with the surgical technique we employed securing the neo-condyle to the anterior lip of the glenoid fossa. Seven of the 15 patients deemed to have anterior displacement greater than 7.5 mm had an average displacement of 11.1 mm (8.3-14.6 mm). We used a cut-off of 5 mm for lateral displacement to still be considered within the glenoid fossa. There were seven patients with lateral displacement; however, only one of them was outside the 5-mm cut-off with a displacement of 10 mm. The lateral displacement subset included patients with destructive squamous cell carcinoma of the mandible, odontogenic cyst, and osteoradionecrosis. The lateral mandibular cortex was involved with disease, or a pathologic fracture was present and prevented template contouring prior to segmental mandibulectomy, likely contributing to a lateral error. In the 21 patients with CT or MRI, despite our findings of anterior and lateral displacement with or without placement in the glenoid fossa, there was no correlation with trismus, crossbite, or resumption of oral diet (Table 1). Three (14.3%) of the patients in the radiographic group had moderate or severe trismus on their postoperative visit, but all returned to normal interincisal distance after the one-year follow-up. Of the six (28.6%) patients who had anterior and lateral displacement together, only one patient did not resume an oral diet. By treating with early mobilization and standardized postoperative evaluation by speech therapy, we were able to identify patients at high risk for severe trismus and provide appropriate additional rehabilitation toward increased interincisal distance.

Concerning partial glossectomy and resumption of an oral diet, of the four patients in our series, two required a G-tube, one died on POD 10, and one returned to an oral diet. Disruption of the native tongue and subsequently the oral phase of food bolus transit would likely interfere with the goals of oral diet. It was noted that eight of the 12 patients who required a cutaneous skin paddle within the oral cavity required a G-tube (66.7%). One of these patients did not return to an oral diet, and two other patients died within the early postoperative period, and therefore their possible advancement to oral diet is unknown. The remaining five patients with a cutaneous skin paddle returned to oral diet within an average of 36 days.

While this study provides valuable insights, it is crucial to acknowledge its limitations to ensure a comprehensive understanding of the research context. The limitations of this study are inherent to its retrospective nature and limited access to certain data points and quality records. The imaging for each patient was taken during different intervals postoperatively and may not be an accurate representation of the patients’ final anatomical outcome. The anterior and lateral displacement values were measured by two different investigators using the measuring tool within the institution's image-viewing software. Additionally, the single-center design may limit the generalizability of the findings, as results may be influenced by institution-specific practices or patient populations. We believe, however, that these limitations are a true reflection of the challenges and complexities intrinsic to managing patients who have undergone segmental mandibulectomy requiring FFF reconstruction. Moving forward, there is a critical need for future studies to enhance the quality of evidence in this field, such as increasing the sample size, to improve statistical power. Despite these limitations, the data presented herein should be considered a valuable addition to the growing body of evidence on the subject.

## Conclusions

It is our experience that when extirpation of the mandibular condyle is required, microvascular reconstruction with fibula free tissue flap is an increasingly viable option. We achieved positive postoperative outcomes in the majority of our patients, alongside the preservation of oral function. It is interesting that despite radiographic evidence of anterior or lateral displacement of neo-condyle, this did not correlate to patients' resumption of oral diet, trismus, or crossbite. Our hospital stays, complications, follow-up, and decannulation rates were all similar or improved compared to the current published literature.
